# Module Analysis Using Single-Patient Differential Expression Signatures Improves the Power of Association Studies for Alzheimer's Disease

**DOI:** 10.3389/fgene.2020.571609

**Published:** 2020-11-20

**Authors:** Jialan Huang, Dong Lu, Guofeng Meng

**Affiliations:** Institute of Interdisciplinary Integrative Medicine Research, Shanghai University of Traditional Chinese Medicine, Shanghai, China

**Keywords:** Alzheimer's disease, single-patient differentially expressed genes, module analysis, SNP, WGCNA

## Abstract

The causal mechanism of Alzheimer's disease is extremely complex. Achieving great statistical power in association studies usually requires a large number of samples. In this work, we illustrated a different strategy to identify AD risk genes by clustering AD patients into modules based on their single-patient differential expression signatures. The evaluation suggested that our method could enrich AD patients with similar clinical manifestations. Applying this to a cohort of only 310 AD patients, we identified 174 AD risk loci at a strict threshold of empirical *p* < 0.05, while only two loci were identified using all the AD patients. As an evaluation, we collected 23 AD risk genes reported in a recent large-scale meta-analysis and found that 18 of them were rediscovered by association studies using clustered AD patients, while only three of them were rediscovered using all AD patients. Functional annotation suggested that AD-associated genetic variants mainly disturbed neuronal/synaptic function. Our results suggested module analysis helped to enrich AD patients affected by the common risk variants.

## 1. Introduction

Alzheimer's disease is a prevalent neurological disease among the aging population. Even with decades of intensive studies, its causal mechanisms remain elusive. Studies of the familial early-onset cases revealed three mutated genes, including APP, PSEN1, and PSEN2 (Lanoiselée et al., 2017). They provided valuable insights into the contribution of the amyloidogenic pathway for AD genesis. Genome-wide association studies (GWAS) of late-onset AD patients discovered more risk genes. Among them, APOE ε4, an apolipoprotein, is a major genetic risk of late-onset AD. It accounts for 3- (heterozygous) to 15-fold (homozygous) increase in AD risk (De Jager et al., 2012). The AlzGene database (http://www.alzgene.org) stores these AD risk genes. Even though many AD risk genes have been discovered, known genes only explain a small proportion of heritability. There is still a great challenge on how to illustrate the AD causal mechanism in an integrated way, limiting their application in drug discovery.

Power is a critical consideration in association studies (Ball, 2013). AD often requires a large sample size to achieve a good power (Belloy et al., 2019; Kunkle et al., 2019). For example, a recent meta-analysis included 71,880 cases and 383,378 controls and identified 25 risk loci, implicating 215 potential causative genes (Jansen et al., 2019). However, such studies are limited by sample collection and cost, which hinders the discovery of more risk variants. To overcome such a problem, a viable strategy is to stratify patients based on some disease-relevant features (Dahl et al., 2019). For AD, the carrier's status of APOE-ε4 had been used to cluster the AD patients in association studies and successfully reported novel features (Marioni et al., 2017). Other factors, e.g., sex (Deming et al., 2018) and age (Lo et al., 2019), have also been used for AD patient stratification and the improved performance supports the values of patient stratification in association studies.

With the increase in multi-omics data, system biology methods show a good potential to unveil the complex mechanism of AD genesis (Wang et al., 2016; Mostafavi et al., 2018; Meng and Mei, 2019). One example is the Accelerating Medicines Partnership–Alzheimer's Disease (AMP-AD) projects, which includes transcriptomics, epigenomics, genetics, and proteomics data from over 2,000 human brain samples. These data allow for AD patient stratification so that the patients affected by the common causal mechanism can be clustered together. Currently, there are some single-patient analysis tools (Vitali et al., 2017). These algorithms integrate gene expression with network or pathway annotation to identify the disease-related changes at a single-patient level (Gardeux et al., 2014; Liu et al., 2014; Schissler et al., 2017). These tools can report disease-relevant genes or pathways at a single-patient level. However, these tools still have some limitations. For example, it is hard to evaluate the generality of reported genes and pathways among different patients, making it less applicable for precise drug discovery.

In this work, we have proposed a new strategy to stratify AD patients by clustering the AD patients with similar differential expression patterns at a single-patient level. Our evaluation suggested that this method could enrich AD patients with common clinical manifestations. We applied it to 310 AD patients for both patient module analysis and genetic association studies. We identified 174 AD risk loci in 143 modules at a strict cutoff of empirical *p* < 0.05, while there were only two risk loci identified using all the AD patients. Function annotation suggested that identified risk genes were mainly related to neuronal/synaptic functions. We also evaluated 23 known AD risk genes and re-discovered 18 of them in at least one module. Allele frequency studies indicated that module analysis using single-patient DEGs enriched AD patients affected by common risk variants.

## 2. Materials and Methods

### 2.1. The Samples and Subjects

The AD and control sample data were collected from the “ROS/MAP” study (De Jager et al., 2012) and “HBTRC” study (Zhang et al., 2013). “ROS/MAP” data included the genotype, expression, and clinical data of 1,788 subjects. The AD-related clinical annotation was provided by the data suppliers. The important one included age, the cognitive score (cts), years of education, ApoE genotype, braak stage (braaksc), and assessment of neuritic plaques (ceradsc). We use the clinical annotation for “cogdx,” a physician's overall cognitive diagnostic category, to select the AD patient (cogdx = 4 or 5) and control subjects (cogdx = 1). After filtering the ones with missing or unclear information for either clinical records or RNA-seq, we found 219 AD patients and 187 control subjects that would be used for module analysis and clinical enrichment studies. “HBTRC” study had both RNA-seq and genotype data for 573 samples, including 311 AD samples. We filtered the one with missed clinical information, RNA-seq, or genotype data. Finally, 310 AD patients and 153 control subjects were used. Detailed information for selected data are publicly available at https://www.synapse.org/#!Synapse:syn5550382.

### 2.2. Clustering AD Patients Using Single-Patient DEGs

We developed a computational algorithm to cluster AD patients (see Supplementary Figure 1). The main idea behind this tool is that AD patients are highly diverse and can be affected by divergent mechanisms; it is possible to cluster AD patients if they shared a subset of differentially expressed genes (DEGs). This algorithm is implemented in the R package DEComplexDisease. It mainly includes four steps:

- Utilize RNA-seq data of normal subjects to construct reference expression profiles. In this step, the parameters of a negative binomial distribution or Gaussian distribution are estimated to describe the distribution profile of non-disease samples;
- The gene expression of AD patients is transformed into binary differential expression status. In this step, the expression values of genes are fitted against reference expression profiles. Binary differential expression status is assigned as 1, –1, or 0 to indicate upregulation, downregulation, or no difference, respectively;
- Apply a bi-clustering analysis to identify DEGs that are repeatedly observed in multiple AD patients, e.g., *n* = 5;
- Using the spDEG of each AD patient as the signature, we compute the co-expression correlation and identify the patients with the most similar expression profiles to construct modules.

The R codes are publicly available at https://github.com/menggf/DEComplexDisease.

### 2.3. Clinical Manifestation Association Analysis

“ROS/MAP” data mainly includes three AD-related clinical features, including cognitive score (cts), CERAD score, and braaksc. “HBTRC” has clinical information for braak and atrophy. Such clinical features can be used to evaluate the disease relevance of modules. Therefore, we applied our tool to generate modules of different sizes, e.g., 40, 60, and 120. For each module, AD patients can be grouped as module patients and non-module patients. We did the Kolmogorov-Smirnov (KS) test to evaluate the clinical manifestation differences between two groups of AD patients.

### 2.4. Processing Genotype Data

We applied stringent quality control (QC) filters to the genotype data. First, we removed the individuals with missing genotype rates >0.05 and SNPs with missing call rate > 0.05. In the next step, the SNPs with minor allele frequency MAF < 0.1 or Hardy-Weinberg equilibrium *p*-value < 1.0 × 10^−5^ were excluded. The individuals with autosomal heterozygosity above empirically determined thresholds were filtered. We calculated the identity-by-descent (IBD) of all possible gene pairs and removed the ones with potential genetic relatedness. These QC filters were performed for multiple rounds to make sure that no individual or SNP could be filtered anyway. We then performed pre-phasing with SHAPEIT2 (Delaneau et al., 2011), using the 1000 Genome Project data as a reference. Afterwards, we conducted whole-genome imputation using IMPUTE2 (Howie et al., 2012) in 5-Mb segments with filtering of the SNP with MAF less than 0.1 in the EUR population. The imputed data were evaluated for quality control using the thresholds mentioned before. We performed principal component analysis (PCA) on autosomal genotype data and adjustment for stratification.

### 2.5. Association Study

Association studies were performed for both AD patients and module patients. To simplify it, we only include the definite AD patients and control individuals in the association analysis so that binary disease status could be assigned for each patient. We performed population stratification by use of the principal components of chromosomal genetic variations. Association analysis was performed using a fast score test implemented in the GenABEL package. In this step, the first 10 principal components were used as covariates to remove the effects of population structure to make sure there was no clear evidence of inflation in the association results. To control the false positive discovery, we also estimated the empirical *p*-values using performing permutation analysis by generating the distribution under the null hypothesis for 1,000 times. In each round of call, the minimal *p*-value was compared with the original *p*-values. For an SNP, its empirical *p*-value is defined as the proportion of times where the minimal *p*-values of 1,000 resamples was less than the original *p*-value. We set empirical *p*-values < 0.05 as the cutoff to selecting the module-associated SNPs. The codes for association studies are available at https://github.com/menggf/spDEG_and_Association.

### 2.6. Enrichment Analysis

We performed enrichment analysis to find the clinical association of patient modules. Among a total of *n* patients, *k* patients were predicted as a module. The number of patients with a clinical manifestation is *p*, which also includes *x* module patients. We used Fisher's exact test to evaluate if the observed *x* patients resulted from random occurrences. We used the following R codes to calculate the p-value:

$$\begin{array}{l} >m=matrix\left(c\left(x,k-x,p-x,n-k\right),ncol=2,byrow=T\right)\\ >p=fisher.test\left(m,alternative=”greater”\right)\$p.value \end{array}$$

## 3. Results

### 3.1. A New Pipeline to Cluster AD Patients Utilizing Single-Patient DEGs

**Figure 1:**
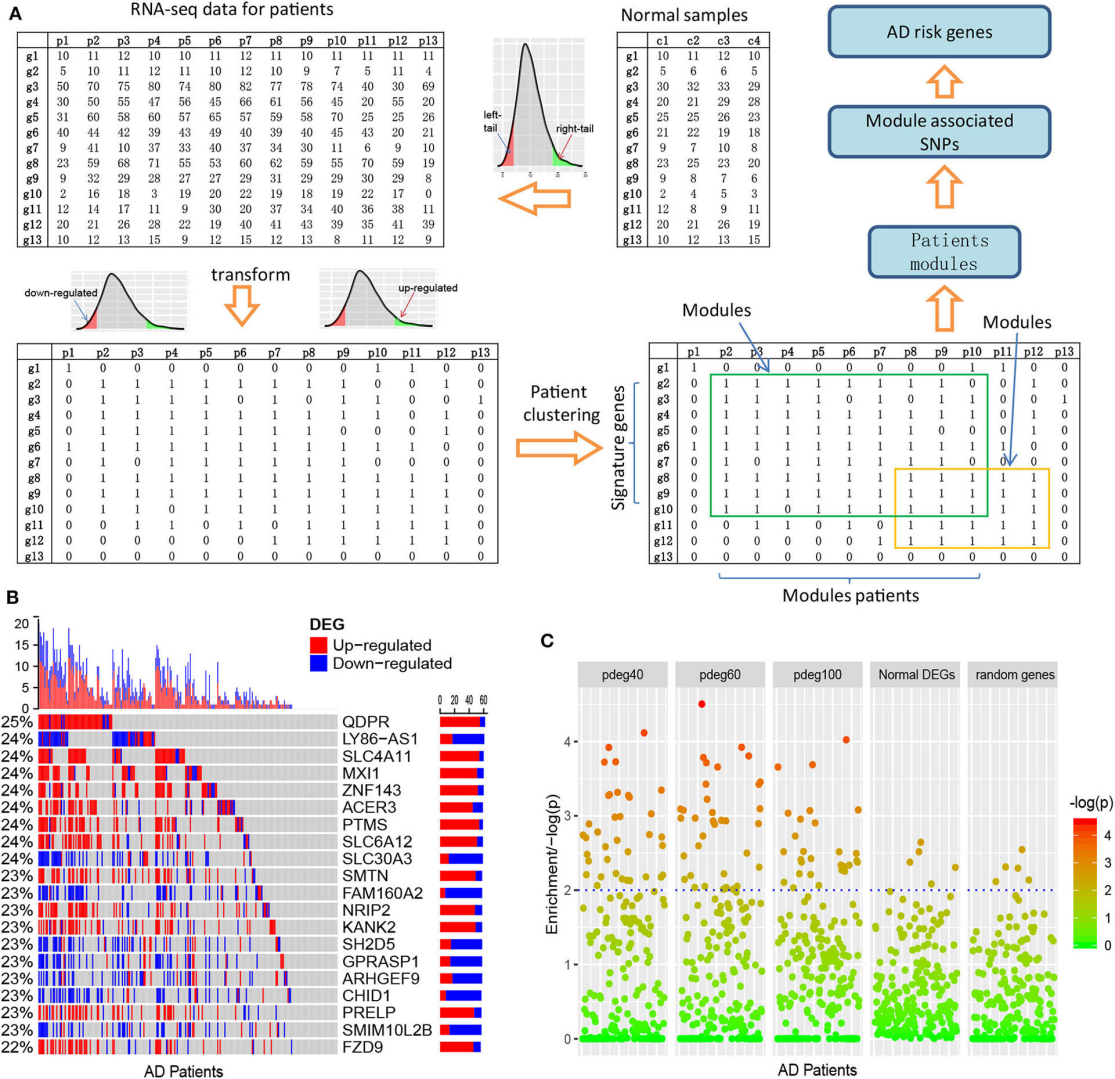
Clustering AD patients into modules based on single-patient differential expression profile similarity. **(A)** An analysis pipeline to cluster AD patients. The RNA-seq data of AD patients were transformed into binary DEG matrix based on the reference profile built using the data of normal individuals; the AD patients with the shared DEG signatures are clustered as modules using a bi-clustering algorithm; genome-wide association study was performed in each patient module to identify the AD risk loci and genes. **(B)** Single-patient differential expression analysis indicated the complexity of AD patients, where genes displayed diverse DE status. **(C)** Module-analysis-enriched AD patients with similar clinical outcomes, e.g., cognitive test scores not by the differentially expressed genes in all AD patients or random genes.

Considering the diversity of AD patients, we propose a new analysis strategy to cluster the AD patients affected by the common mechanisms. This method is based on differential expression analysis at single-patient levels. Figure 1A and Supplementary Figure 1 describe the schema of the whole analysis pipeline. In our analysis, the reference expression profile was firstly built using the RNA-seq counts or normalized data of the normal individuals, which defined the ranges of gene expression values at a non-disease status. Next, gene expression values of patients were transformed into binary status by fitting to the reference expression profiles. In detail, if the gene expression values of patients exceeded the range of reference expression profiles, 1 or –1 is assigned to indicate up- or downregulation. To improve confidence, a bi-clustering analysis algorithm is applied to perform filtering and cross-validation so that the whole set of *single-patient differentially expressed genes* (spDEGs) can be repeatedly observed in multiple patients, e.g., *n* = 5. Finally, using each patient as a seed, we cluster the patients into modules if they carry the same set of spDEGs.

As an evaluation, we applied this pipeline to the dataset collected from the ROS/MAP study (De Jager et al., 2012), which includes 251 AD samples with both RNA-seq data and clinical annotation. We identified cross-validated spDEGs for 171 patients. Among 15,582 brain expressed genes, 3,878 spDEGs were predicted to be differentially expressed in at least one AD patient at a cutoff of *p* < 0.05. Compared to traditional differential expression analysis using all the AD samples, they covered 93.8% of AD DEGs. We then investigated their differential expression status among all the AD patients. Figure 1B showed the results of the top 20 most observed spDEGs. We did not observe any shared differential expressed genes across all the AD patients. On the contrary, all spDEGs were only differentially expressed in a small proportion of 251 AD patients. Additionally, we also observed inconsistent differential expression directions. Taking the QDPR gene as an example, it was upregulated in 22% of AD patients while also downregulated in 3% AD patients. Similar results were observed with other spDEGs (see Figure 1B). We also performed module analysis using the most observed differential expressed genes and observed distinct differential expression patterns (see Supplementary Figures 2, 3). All these results suggested that AD patients were greatly diverse and that it would be a risk to treat AD patients as a homogeneous whole in any analysis.

Next, we investigated if AD patient clustering could enrich AD patients with common clinical manifestations. We generated patient modules based on spDEG expression profile similarity. The modules were arbitrarily set to have different sizes of AD patients, e.g., 40, 60, and 100, which could be denoted as pdeg40, pdeg60, and pdeg100, respectively. The patients within the same module were supposed to be affected by the common mechanisms. As a control, we also generated modules using randomly selecting genes and DEGs identified by traditional differential expression analysis. Figure 1C showed the evaluation results using cognitive scores (cts). At a cutoff of *p* < 0.01, 37 “pdeg60” modules were enriched with detrimental cts scores, while only five modules identified by common DEGs or random genes were enriched. The most significant *p*-value was up to *p* = 2.51 × 10^−5^ in the “pdeg60” module. On the contrary, no module in “common DEG” and "random gene" exceeded the significance of *p* = 0.001. This result suggested that modules analysis using spDEG better-enriched AD patients with common clinical manifestations.

### 3.2. More Risk Variants Were Identified in AD Patient Modules

**Figure 2:**
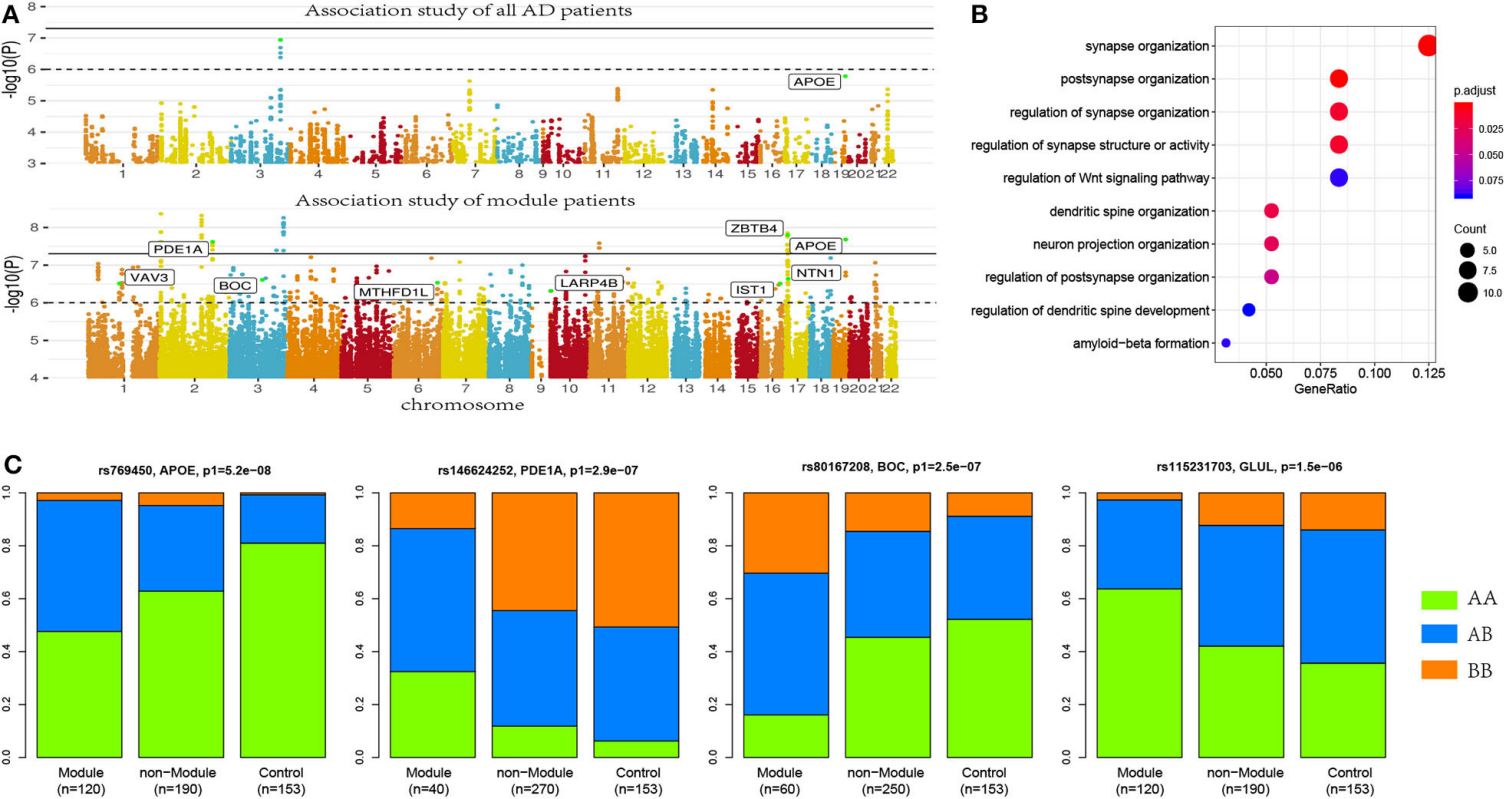
More risk variants were identified in AD patient modules. **(A)** Manhattan plot for the association studies to both AD patients and patient modules, where more AD risk SNPs were identified in AD modules. **(B)** Allele frequencies in module patients, non-module patients, and control subjects. More risk allele enrichment was observed in module patients, suggested that module analysis enriched the AD patients affected by common risk variance. **(C)** Functional annotation to AD risk genes. Here, synaptic-function-related terms were most significantly enriched.

We collected genotyping data from the “hbtrc” study (Zhang et al., 2013), including 310 LOAD patients and 153 non-demented healthy controls. We performed genome-wide association studies (GWAS) using all the AD patients. In this process, we performed a permutation procedure for 1,000 times to estimate empirical *p* values. We found only two loci to have a significant association with AD at a cutoff of empirical *p* < 0.05. The significant SNPs included rs2405283 (*p* = 1.15 × 10^−7^) and rs769450 (*p* = 1.65 × 10^−6^) (see Figure 2A). rs769450 was mapped to the second intron of the APOE gene, which is consistent with published reports about the critical roles of APOE.

Applying module analysis, we predicted 143 modules of AD patients. Three association tests were performed for each module: (1) module patients against normal control; (2) module patients against non-module patients; and (3) non-module patients against normal control. The *p*-values were denoted as *p*1, *p*2, and *p*3, respectively. At a strict cutoff of empirical *p*1 < 0.05, we found 174 loci to have a significant association in at least one of 143 modules (see Figure 2A and Supplementary Table 1). Compared to the results of the association study using all the AD patients, more AD risk loci were observed within module patients. The APOE SNP rs769450 was observed in 41 modules and its association significance was also greatly improved. For example, the significance of rs769450 was up to *p*1 = 2.08 × 10^−8^ in a module of 80 AD patients while the significance for all 310 patients was *p*1 = 1.65 × 10^−6^. Tests between module patients and non-module patients supported allele frequency differences in 165 out of 174 loci at a cutoff of *p*2 < 0.01. Figure 2B showed the allele frequency for some exemplary SNPs. We observed that allele frequencies of identified risk SNPs were different from the non-module patients and normal individuals. In most cases, non-module patients usually had similar allele frequencies with normal subjects. We checked if module patients were more associated with risk SNPs than non-module patients by comparing *p*1 and *p*3 value distribution (see Supplementary Figure 4). We found module patients tended to report more significant associations than non-module patients. It suggested that module analysis enriched the AD patient affected by the common risk SNPs.

We mapped 174 AD risk loci to 107 genes based on genomic proxy and GTEx eQTL annotation (see Supplementary Table 1). Among them, 86 genes were observed in more than one module at a cutoff of empirical *p*1 < 0.05. APOE is the most observed risk gene, which is significantly associated with AD patients in 41 modules. We searched the published GWAS results and found that 46 genes had been reported for AD or brain-related function (see Table 1). Some of them had been reported in association studies of AD, such as PDE1A, JAM3, DLGAP1, CYYR1, SERPINB11, and MCPH1. To understand their function involvement, we performed Gene Ontology enrichment analysis to 107 AD risk genes (see Figure 2C). We found that the most enriched terms were also related to synaptic and neuronal function, e.g., “synapse organization” (*p* = 7.65 × 10^−6^). It suggested that the identified AD risk genes were related to normal brain function and had potential roles in AD genesis.

**Table 1:** The results of association studies using module patients, non-module patients, and control.

**id**	**chr**	**pos**	**p1**	**p2**	**p3**	**p(emp)**	**OR**	**Type**	**Gene**	**Region**	**PubMed ID**
rs3867593	17	7464046	1.59E-08	1.20E-04	3.73E-03	0.001	2.63	pdeg60	ZBTB4	Intron	29045054
rs769450	19	44907187	2.08E-08	1.94E-02	1.44E-04	0.001	2.91	pdeg80	APOE	Intron	24821312
rs146624252	2	182412080	2.40E-08	3.14E-07	2.60E-01	0.002	2.01	pdeg60	PDE1A	Intron	29363967
rs9912864	17	9105233	2.31E-07	9.05E-04	2.53E-02	0.005	2.51	pdeg120	NTN1	Intron	27060954
rs80167208	3	113224966	2.46E-07	1.69E-05	1.11E-01	0.009	1.99	pdeg60	BOC	Intron	22445332
rs34233526	6	150947695	2.91E-07	6.86E-06	5.81E-02	0.013	2.31	pdeg60	MTHFD1L	Intron	22330827
rs72129870	1	107645322	3.07E-07	1.25E-05	3.32E-01	0.008	2.03	pdeg100	VAV3	Intron	28927664
rs4788579	16	71917942	3.77E-07	3.00E-04	1.45E-02	0.011	3.24	pdeg60	IST1	Intron	31223056
rs113337484	6	87710980	4.05E-07	5.46E-04	2.03E-02	0.009	2.87	pdeg60	AKIRIN2	Intergenic	27871306
rs11253483	10	872071	4.83E-07	7.06E-04	5.34E-03	0.029	2.26	pdeg40	LARP4B	Intron	20435134
rs17077094	8	6480005	5.50E-07	3.75E-03	1.73E-02	0.015	3.01	pdeg60	MCPH1	Intron	21297427
rs11339072	11	85061332	5.87E-07	4.92E-04	2.66E-02	0.019	2.86	pdeg60	DLG2	intron	29290481
rs33954745	2	169259162	6.69E-07	2.54E-04	8.27E-02	0.039	0.52	pdeg60	LRP2	Exon	20971101
rs11412426	3	65493079	6.77E-07	8.09E-04	1.07E-02	0.012	0.44	pdeg80	MAGI1	Intron	22166940
rs222960	21	26551898	7.11E-07	2.34E-04	4.81E-02	0.006	2.19	pdeg80	CYYR1	Intron	30820047
rs8088835	18	3728055	7.17E-07	6.90E-05	1.76E-01	0.006	3.23	pdeg120	DLGAP1	Intron	30448613
rs11859292	16	6491819	8.68E-07	7.55E-03	3.35E-03	0.02	2.09	pdeg80	RBFOX1	NMD	30596066
rs10138555	14	30020759	8.74E-07	2.14E-04	2.10E-02	0.035	2.15	pdeg60	PRKD1	Nocoding	21696630
rs2501215	13	70069895	9.29E-07	1.92E-03	1.76E-02	0.011	2.47	pdeg100	KLHL1	Intron	15715669
rs1783749	11	85049683	9.82E-07	4.92E-04	3.71E-02	0.03	4.01	pdeg60	DLG2	Intron	29290481
rs348658	12	62063579	1.04E-06	2.38E-03	2.33E-02	0.028	3.06	pdeg80	TAFA2	Intron	30137205
rs6958644	7	139796416	1.06E-06	1.91E-03	1.92E-02	0.035	2.03	pdeg80	TBXAS1	Nocoding	24608097
rs5892206	8	69583407	1.11E-06	2.10E-02	1.89E-03	0.04	2.67	pdeg60	SULF1	Intron	30035253
rs11862587	16	83628162	1.27E-06	2.69E-04	2.01E-02	0.021	2.11	pdeg60	CDH13	Intron	29771432 26460479
rs28764186	17	79306443	1.30E-06	6.08E-03	8.46E-03	0.021	0.44	pdeg100	RBFOX3	Intron	30475774
rs12281243	11	40133562	1.46E-06	6.98E-05	9.56E-02	0.039	2.58	pdeg60	LRRC4C	Intron	29751835
rs12705741	7	110873688	1.48E-06	2.33E-04	1.18E-01	0.046	2.14	pdeg80	IMMP2L	Intron	22486522
rs2373961	7	150984122	1.50E-06	1.45E-07	8.23E-01	0.044	0.42	pdeg80	KCNH2	Intergenic	19412172
rs115231703	1	182348704	1.51E-06	5.15E-05	3.88E-01	0.046	0.47	pdeg120	GLUL	Intergenic	29441491
rs548084743	17	47919005	1.64E-06	4.09E-04	1.29E-01	0.028	2.26	pdeg60	SP2 SP2-AS1	Intron	23293287
rs77144903	13	102144657	1.82E-06	1.41E-03	3.92E-02	0.039	0.2	pdeg100	FGF14	Intron	28522250 28469558
rs146092846	15	100217974	1.87E-06	1.22E-03	4.53E-02	0.03	0.43	pdeg120	ADAMTS17	Intron	22710270
rs7147828	14	71994665	1.88E-06	7.58E-04	8.16E-02	0.039	2.18	pdeg80	RGS6	Intron	27002730
rs75538719	8	36794270	1.90E-06	3.36E-03	8.00E-03	0.046	2.47	pdeg100	KCNU1	Intron	26858991
rs2977548	8	133224849	1.92E-06	7.50E-04	1.05E-01	0.044	2.53	pdeg60	CCN4	NMD	22475393
rs78818922	14	54638870	2.03E-06	5.89E-04	1.87E-01	0.038	2.06	pdeg100	SAMD4A	Intron	29432188
rs62223372	21	31377966	2.04E-06	1.09E-03	8.62E-03	0.009	0.42	pdeg80	TIAM1	Intron	23109420
rs12881844	14	51639930	2.06E-06	1.79E-02	1.77E-03	0.023	0.39	pdeg120	FRMD6	Nocoding	22190428
rs609214	13	102174932	2.20E-06	3.00E-03	7.04E-02	0.037	0.23	pdeg120	FGF14	Intron	28522250 28469558
rs4903566	14	77274080	2.30E-06	9.77E-05	9.71E-02	0.045	0.46	pdeg60	POMT2	Intergenic	22984654
rs60119577	18	57155356	2.56E-06	1.22E-03	1.59E-01	0.029	0.41	pdeg100	BOD1L2	Intergenic	27166630
rs146623074	15	32107801	2.76E-06	2.36E-03	6.07E-03	0.035	0.43	pdeg80	CHRNA7	Intron	24951635
rs141887840	18	79482278	2.79E-06	1.18E-03	3.07E-02	0.036	2	pdeg60	NFATC1	Intron	20401186
rs12902710	15	55318928	3.03E-06	7.21E-04	6.12E-02	0.048	0.47	pdeg100	PIGBOS1 RAB27A	5'UTR	26985808
rs10444855	15	33393629	3.25E-06	1.97E-07	5.52E-01	0.047	1.89	pdeg60	RYR3	Intron	29590321
rs6103379	20	43547767	3.94E-06	2.28E-04	1.99E-01	0.041	0.49	pdeg100	L3MBTL1	NMD	29898393 31061493

In a recent large-scale meta-analysis, 23 AD risk loci were reported (Kunkle et al., 2019). We checked their association using either all patients or module patients. We loosed the cutoffs of significant association by replacing empirical *p* < 0.05 with *p*1 < 10^−4^. Association study using all AD patients failed to identify any extra known AD risk gene to satisfy a threshold of *p*1 < 10^−4^. Unlike the results using all AD patients, we observed that 18 out of 23 AD genes have a significant association with AD in at least one module. Table 2 summarized the analysis results using module patients. By checking *p*2 and *p*3 values, we found significant allele frequency differences between module patients and no-module patients, supporting the conclusion that module analysis enriched AD patients affected by commonly known risk variants.

**Table 2:** The association results for known AD risk genes.

**Association of module patients**									**Association of all AD patients**			
**SNP**	**gene**	**p1**	**p2**	**p3**	**p(emp)**	**OR**	**Module type**	**Region**	**SNP**	**p1**	**p(emp)**	**Region**
rs769450	APOE	2.08E-08	1.94E-02	1.44E-04	0.001	3.68	pdeg80	Intron	rs769450	1.65E-06	0.015	Intron
rs71454394	MS4A2	9.25E-06	3.73E-03	4.32E-02	0.257	2.48	pdeg40	Intergenic	–	–	–	–
rs9462659	TREM2	1.08E-05	8.99E-03	4.85E-02	0.35	2.02	pdeg40	Intergenic	–	–	–	–
rs7152488	SLC24A4	1.21E-05	1.85E-04	1.71E-01	0.175	0.3	pdeg100	Intron	–	–	–	–
rs5021727	HLA-DRB1	1.59E-05	1.80E-04	3.88E-01	0.389	0.45	pdeg120	Intergenic	–	–	–	–
rs144409358	CR1	2.09E-05	1.44E-03	1.79E-01	0.552	0.3	pdeg120	Intron	–	–	–	–
rs12416009	ECHDC3	2.10E-05	2.66E-04	2.19E-01	0.514	1.86	pdeg40	Intergenic	–	–	–	–
rs9897336	ACE	2.41E-05	2.03E-04	4.91E-01	0.306	0.48	pdeg100	Intergenic	–	–	–	–
rs55662472	EPHA1	2.61E-05	5.33E-03	7.65E-02	0.519	3.15	pdeg80	Intergenic	–	–	–	–
rs34708229	MEF2C	2.81E-05	4.09E-03	2.17E-02	0.675	2.45	pdeg40	Intron	rs79820174	1.40E-04	1	Intron
rs6099038	CASS4	2.86E-05	1.55E-04	3.16E-01	0.305	2.30	pdeg100	Intergenic	–	–	–	–
rs13422890	BIN1	3.35E-05	4.42E-06	8.04E-01	0.753	1.96	pdeg60	Intron	–	–	–	–
rs36057699	PTK2B	3.39E-05	8.08E-03	4.63E-02	0.576	0.41	pdeg120	Intron	rs36057699	8.70E-04	1	intron
rs659023	PICALM	6.53E-05	8.73E-06	4.35E-01	0.797	0.54	pdeg120	Intergenic	–	–	–	–
rs77792633	FERMT2	8.95E-05	5.18E-04	5.44E-01	0.8	0.62	pdeg60	Intergenic	–	–	–	–
rs57816367	CD2AP	9.17E-05	9.36E-05	4.60E-01	0.957	2.13	pdeg40	Intron	–	–	–	–
rs10539341	INPP5D	9.42E-05	7.99E-03	9.36E-02	0.983	0.42	pdeg100	Intron	–	–	–	–
rs2285898	ABCA7	9.09E-05	1.00E-02	1.48E-01	0.632	0.53	pdeg120	Intergenic	–	–	–	–

### 3.3. Biological Relevance of AD Risk Genes

Module-based clustering analysis allows us to bridge AD risk genes to clinical features and affected biological processes. The clinical association of modules is determined by enrichment analysis. In HBRTC's dataset, we identified nine and eight modules to be associated with braak and brain generalized atrophy at a cutoff of *p* < 0.01, respectively. Among them, three modules were associated with both braak and brain atrophy. Association study to these modules identified eight and 20 loci respectively. In Table 3, we summarized the analysis results. These results supported that some AD risk genes might be more associated with some AD clinical outcomes. For example, the NTN1 gene is a microtubule-associated force-producing protein and it is predicted to be related to the braak stage.

**Table 3:** The association results for known AD risk genes.

**SNP**	**chr**	**pos**	**p1**	**p1(emp)**	**Seed patient**	**tp**	**Gene**	**Region**	**Braak**	**Atrophy**
**SNPs ASSOCIATED WITH ATROPHY**										
rs147216627	1	157467609	7.04E-07	0.022	X15888	pdeg100	–		7.4E-02	9.05E-04
rs78818922	14	54638870	2.03E-06	0.038	X15888	pdeg100	SAMD4A	Intron	7.4E-02	9.05E-04
rs1231702	11	29525814	4.97E-07	0.012	X15914	pdeg60	AC110058.1,AC090124.1	Intergenic	0.95	7.96E-03
rs3867263	18	63664376	9.40E-07	0.031	X15914	pdeg40	SERPINB11	Intron	0.24	7.18E-03
rs236111	20	5952889	7.13E-07	0.011	X15914	pdeg60	MCM8	Intron	0.95	7.96E-03
rs7113161	11	16969038	9.68E-07	0.024	X15941	pdeg120	PLEKHA7	Intron	1.34E-02	3.82E-03
rs10489293	1	172217647	1.12E-07	0.005	X16020	pdeg40	DNM3	Intron	6.52E-02	7.87E-04
rs12819631	12	104013393	2.84E-07	0.01	X16020	pdeg40	GLT8D2	Intron	6.52E-02	7.87E-04
rs9912864	17	9105233	2.89E-06	0.037	X16020	pdeg100	NTN1	Intron	0.97	2.28E-03
rs6875561	5	121537532	1.47E-06	0.049	X16037	pdeg80	–		1.27E-02	1.42E-03
rs7930638	11	5567722	1.85E-06	0.043	X16179	pdeg120	AC104389.4	NMD	3.56E-02	4.16E-03
rs548084743	17	47919005	9.62E-07	0.021	X16179	pdeg40	SP2,SP2-AS1	Intron	9.51E-02	7.26E-03
rs764624	14	71993857	2.32E-06	0.049	X16183	pdeg60	RGS6	Intron	0.11	8.66E-03
rs78641850	10	53421383	2.17E-07	0.001	X21821	pdeg100	–		2.02E-02	6.43E-03
rs17112518	14	21948703	2.30E-06	0.027	X21901	pdeg120	–		0.10	6.98E-03
rs12881844	14	51639930	2.06E-06	0.023	X21901	pdeg120	FRMD6	Intergenic	0.10	6.98E-03
rs12480378	20	3110711	2.29E-06	0.025	X21901	pdeg120	UBOX5-AS1,UBOX5	Intergenic	0.10	6.98E-03
**SNPs ASSOCIATED WITH BRAAK**										
rs6103379	20	43547767	3.94E-06	0.041	X15917	pdeg100	Z98752.3,L3MBTL1	NMD	8.37E-03	1.01E-01
rs11850894	14	22312243	2.04E-06	0.033	X15989	pdeg80	TRAV40	Intergenic	1.33E-04	7.33E-02
rs73699762	7	57341624	1.01E-06	0.028	X15989	pdeg120	–		2.55E-03	6.59E-02
rs222960	21	26551898	4.39E-06	0.033	X16038	pdeg60	CYYR1,CYYR1-AS1	Intron	7.20E-03	5.03E-01
rs6880404	5	163990493	9.32E-07	0.031	X16105	pdeg120	–		2.86E-03	4.77E-02
rs538060878	17	9142309	1.10E-07	0.004	X21810	pdeg40	NTN1	Intron	4.27E-03	6.56E-01
rs1016268	12	129517265	1.88E-06	0.048	X21810	pdeg80	TMEM132D	Intron	6.58E-04	1.92E-01
rs6769967	3	44217312	1.77E-07	0.012	X21810	pdeg40	–		4.27E-03	6.56E-01
rs16885931	6	22265940	8.04E-07	0.021	X21810	pdeg120	CASC15	Intergenic	6.11E-04	2.32E-02
**SNPs ASSOCIATED WITH BOTH ATROPHY AND BRAAK**										
rs769450	19	44907187	7.12E-07	0.008	X16149	pdeg120	APOE	Intron	9.62E-03	1.94E-03
rs78415808	12	69406115	8.07E-07	0.023	X16183	pdeg80	–		2.18E-03	5.84E-03
rs820562	3	112745366	1.46E-06	0.042	X16037	pdeg120	LINC02042	Intergenic	6.11E-03	3.99E-05

**Figure 3:**
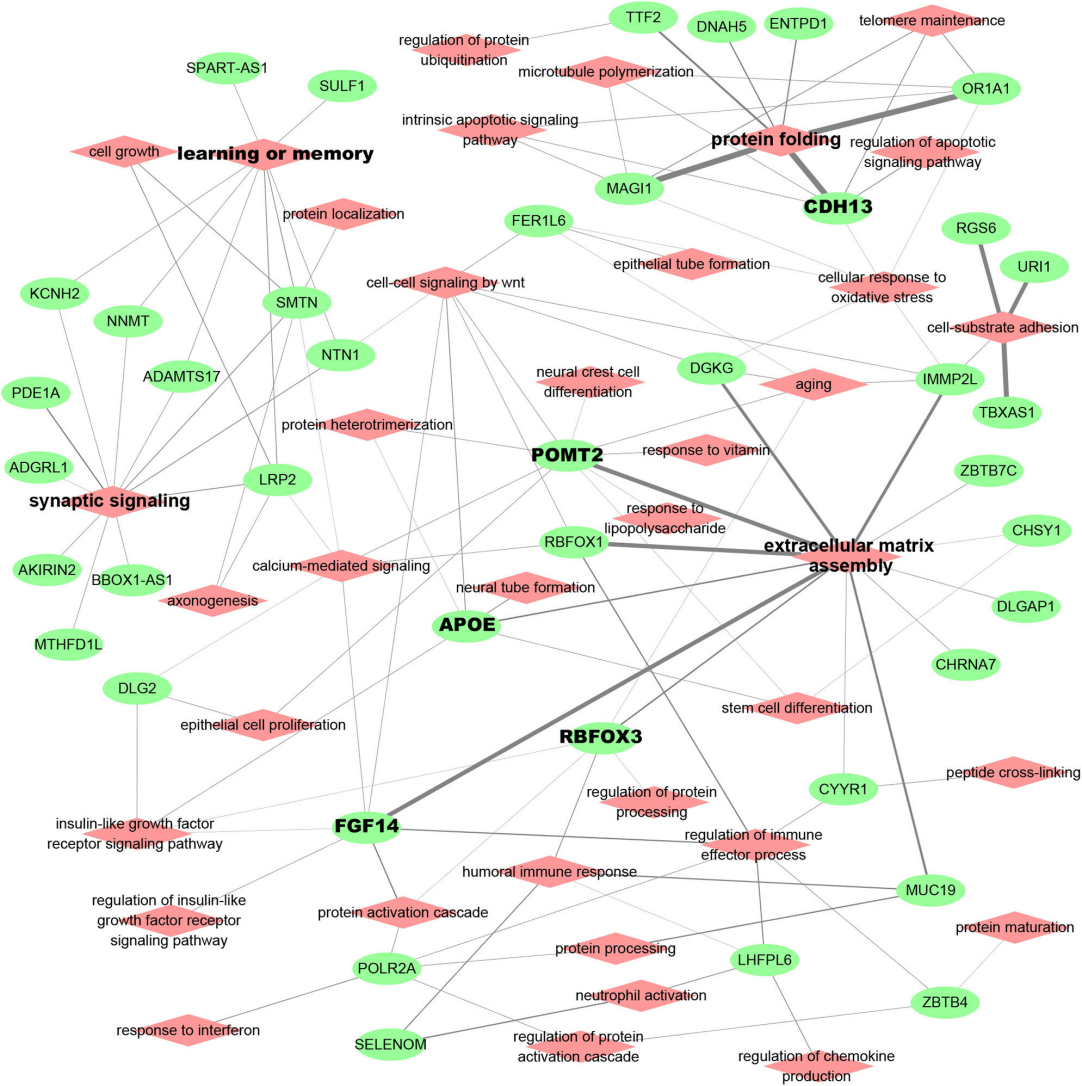
Functional relevance of AD risk genes. Here, the module spDEG signatures were used for Gene Ontology enrichment to indicate the functional involvement of modules.

AD patient modules are always associated with a list of spDEG signature genes, which could be used to investigate the biological relevance of AD risk genes. Figure 3 showed the analysis results of functional annotation to module spDEG signature genes. Among the significant terms, "extracellular matrix assembly," "synaptic signaling," "learning and memory," and "protein folding" were more observed or more significant. By text mining studies, we found much published evidence for their close association with AD, supporting that predicted AD risk genes contributed to AD development. For example, the extracellular matrix was observed to have significant changes during the early stages of AD (Lepelletier et al., 2017) and extracellular matrix could induce β-Amyloid Levels (Ma et al., 2019). Among predicted risk genes, APOE, POMT2, FGF14, CDH13, and RBFOX3 display more functional involvements.

### 3.4. Evaluation Using Randomly Modules of AD Patients

In the above analysis, we attempted to cluster AD patients with a common set of spDEGs so that the clustered patients were more affected by common AD variants. As an evaluation, we performed a simulated study by randomly splitting AD patients into simulated modules at corresponding sizes. We then predicted AD risk SNPs using the same setting. In each round of the simulation, we identified about 105 AD risk SNPs on average at a cutoff of empirical *p* < 0.05. We compared their analysis results to those of true modules and found that about 63% of risked SNPs (out of total 174 loci) could be overlapped with the SNPs predicted using true modules. This evaluation seemed to support the conclusion that subsetting AD patients had benefits to improve the power of association studies, even when the criteria to stratify AD patients was to randomly pick up. Compared to random modules, modules of spDEG signature could recover more AD risk SNPs.

## 4. Discussion

In this work, we took more consideration to AD patient diversity and attempted to stratify patients into modules affected by different genetic background. We therefore came up with an analysis pipeline to cluster AD patients based on some assumptions. These included that (1) AD patients are very diverse and differential expression patterns differ among AD patients, and that (2) we can use single-patient DEGs as biomarkers to indicate the dysregulation status of AD patients and to cluster the AD patients affected by common mechanisms. In our previous work, we have applied similar strategies to discover enriched transcription factor binding sites (Meng and Vingron, 2014) and cancer driver mutations (Meng, 2018), and we achieved a good performance. Evaluation using real patient data suggested that this method could group AD patients with similar clinical outcomes and common risk variants, validating our assumptions. Compared to existent methods, our pipeline not only can discover patient-specific DEGs but also considers the reliability of spDEGs by evaluating their occurrence in multiple patients.

We applied it to find the differentially expressed genes for each AD patient and module patients based on the spDEG signatures. In this process, we made some assumptions. For example, we defined the reference expression profiles for normal individuals by fitting to a Gaussian or negative binomial distribution. The robustness of this step was dependent on the number and homogeneity of control individuals. To identify the differentially expressed genes, we need to set some thresholds to determine if the gene expression level of one AD patient was beyond the normal ranges. In our work, we tested different cutoffs and selected *p* = 0.1.

We did an association study in each module of size 40 to 120. Compared to the study using all AD patients, the statistical power decreased with a decreased sample size in each association study. However, more AD risk loci were identified for the increased number of AD patient modules. A total of 174 loci were predicted to be associated with AD at a strict threshold of empirical *p* < 0.05, while only two loci exceed such a threshold using all AD patients. The genotype frequency was found to be different between module and non-module patients. All these results suggested that AD risk variants might contribute only a limited subset of the AD population.

During simulation analysis, we also predicted many AD risk SNPs. The reason could be that random sampling also enriched AD patients affected by some AD risk SNPs. For example, we found APOE SNPs were not associated with AD patients in nearly half of simulated modules. It suggested that randomly sampling enriched the AD patient less affected by APOE. Similarly, it was possible to enrich the AD patients affected by other AD risk SNPs, especially when the AD sample size was limited.

As an evaluation, we also collected ROSMAP data and performed a similar study. We found that our method helped to identify more AD risk genes, validating our conclusion that module analysis improved the power of association study. However, we observed only limited overlaps for identified AD risk SNPs between ROSMAP and HBTRC dataset (see Table 4). The reasons could be that (1) there were only 251 AD patients in ROSMAP data, which were too limited to recover full AD risk SNPs, and (2) the cutoff of the association study was too strict to identify all the AD risk SNPs.

**Table 4:** The association results for ROSMAP data.

**id**	**chr**	**pos**	**p1**	**p2**	**p3**	**p(emp)**	**Type**	**Gene**
rs2688748	10	70744565	9.85E-08	1.51E-06	–	0.04	pdeg60	ADAMTS14
rs2532019	16	4074299	3.60E-08	5.80E-05	–	0.03	pdeg40	ADCY9
rs117051234	8	6538298	2.56E-07	–	–	0.025	pdeg120	ANGPT2,MCPH1
rs118150738	12	99429000	7.79E-09	2.32E-07	–	0.025	pdeg40	ANKS1B
rs1064725	19	44919304	1.20E-09	–	–	0.005	pdeg40	APOC1
rs75524475	9	33021586	1.01E-07	–	–	0.03	pdeg60	APTX
rs55791516	14	58299182	8.04E-08	1.81E-05	–	0.005	pdeg60	ARID4A
rs79038737	16	80841428	2.24E-09	–	–	0.005	pdeg60	ARLNC1
rs8033014	15	50144866	6.64E-07	2.48E-06	–	0.04	pdeg60	ATP8B4
rs2154498	21	29311727	6.36E-07	–	–	0.04	pdeg40	BACH1
rs230107	9	119163787	1.91E-08	1.22E-06	–	0.005	pdeg80	BRINP1
rs11641442	16	83320409	5.49E-08	–	–	0.005	pdeg80	CDH13
rs142424916	10	60780861	8.23E-09	9.85E-06	–	0.005	pdeg40	CDK1
rs117661233	14	95269426	6.51E-07	–	–	0.03	pdeg120	CLMN
rs7723296	5	10306906	5.66E-08	–	–	0.01	pdeg60	CMBL
rs142513159	18	52370108	6.14E-08	9.24E-09	–	0.03	pdeg40	DCC
rs10971346	9	33031085	6.41E-08	–	–	0.02	pdeg60	DNAJA1
rs16990792	22	43593673	1.44E-08	–	–	0.005	pdeg100	EFCAB6
rs148526127	14	99756716	4.68E-09	–	–	0.004975124	pdeg60	EML1
rs7279562	21	32036384	2.85E-06	–	–	0.04	pdeg120	HUNK
rs79334679	3	124548449	1.99E-08	1.50E-08	–	0.01	pdeg60	KALRN
rs73423776	7	120689401	1.77E-07	1.19E-06	–	0.025	pdeg60	KCND2
rs150056741	10	77390525	4.25E-09	7.91E-06	–	0.004975124	pdeg60	KCNMA1
rs11654934	17	40978630	2.05E-08	5.95E-08	–	0.01	pdeg60	KRT40
rs140730427	17	41041338	5.90E-08	2.91E-05	–	0.015	pdeg60	KRTAP1-1
rs11792940	9	33019794	1.52E-07	–	–	0.04	pdeg60	APTX
rs3730850	19	48165452	2.85E-07	1.26E-05	–	0.025	pdeg40	LIG1
rs986117	18	46548540	5.90E-07	–	–	0.035	pdeg100	LOXHD1
rs112704814	2	141324888	1.33E-08	3.94E-05	–	0.005	pdeg120	LRP1B
rs73193820	21	29172348	1.39E-06	2.09E-06	–	0.01	pdeg80	MAP3K7CL
rs73539906	6	110302890	1.87E-10	1.31E-05	–	0.005	pdeg40	METTL24
rs7871013	9	75146220	1.27E-08	3.16E-06	–	0.025	pdeg40	OSTF1
rs10151276	14	57051817	3.32E-07	–	–	0.02	pdeg80	OTX2-AS1
rs11564502	19	48110444	5.88E-08	7.32E-05	–	0.005	pdeg80	PLA2G4C
rs41278865	22	43880927	1.92E-08	5.81E-05	–	0.005	pdeg40	PNPLA5
rs4448724	12	27578227	2.11E-07	1.37E-06	–	0.04	pdeg60	PPFIBP1
rs34304517	12	62739081	8.98E-08	3.88E-06	–	0.025	pdeg40	PPM1H
rs230159	20	42487156	2.85E-07	4.12E-05	–	0.015	pdeg100	PTPRT
rs80087065	14	68520366	2.10E-08	2.30E-08	–	0.005	pdeg40	RAD51B
rs17242783	14	20695527	5.76E-09	–	–	0.005	pdeg40	RNASE4
rs10925501	1	237738307	2.84E-07	–	–	0.015	pdeg120	RYR2
rs111671818	14	71543950	7.48E-07	–	–	0.005	pdeg120	SIPA1L1
rs2274766	9	33055812	8.97E-08	–	–	0.03	pdeg60	SMU1
rs34601004	22	43861694	2.15E-08	2.18E-05	–	0.005	pdeg40	SULT4A1
rs56171440	6	158715662	4.29E-15	6.66E-06	–	0.005	pdeg40	SYTL3
rs141418488	4	182544545	3.03E-08	2.20E-05	–	0.005	pdeg60	TENM3
rs73571693	6	155057540	1.31E-07	2.25E-06	–	0.045	pdeg60	TIAM2
rs4985720	17	16958916	1.74E-06	–	–	0.04	pdeg120	TNFRSF13B
rs4235957	6	158250454	1.91E-08	5.56E-07	–	0.01	pdeg60	TULP4
rs16949592	16	79123857	1.95E-07	–	–	0.035	pdeg60	WWOX
rs11053909	12	10703084	2.92E-08	4.14E-06	–	0.03	pdeg40	YBX3
rs11100901	4	145825808	2.91E-08	–	–	0.04	pdeg40	ZNF827

In this work, we proved the benefits of the patient module in association studies to AD. In our application, we reported more AD risk genes even when only 310 AD patients were used. In the large-scale meta-analysis, there were about 20–30 genes identified as AD risk genes (Cuyvers and Sleegers, 2016; Jansen et al., 2019). However, by searching public literature and databases, e.g., the GWAS catalog, we found more than 100 studies and 300 genes that had been reported in associated studies to AD patients. These studies could be treated as a subset of large-scale AD meta-analysis. This result suggested that there might be more AD risk genes, and AD patient subsetting helped to identify them.

## Data Availability Statement

Publicly available datasets were analyzed in this study. This data can be found at: https://www.synapse.org/#!Synapse:syn2580853.

## Author Contributions

GM designed the project. JH and DL contributed to collected samples and analyzed the data. JH and DL contributed to analyzed results and wrote the manuscript. JH, DL, and GM revised the manuscript. All authors read and approved the final manuscript.

## Conflict of Interest

The authors declare that the research was conducted in the absence of any commercial or financial relationships that could be construed as a potential conflict of interest.
