# Supplementary material for: Module Analysis Using Single-Patient Differential Expression Signatures Improves the Power of Association Studies for Alzheimer's Disease
Source: Front Genet. 2020 Nov 20;11:571609. doi: 10.3389/fgene.2020.571609 (PMC7714954; doi:10.3389/fgene.2020.571609)
Supplement: Supplementary file 2 [file Presentation_1.PDF]

# Supplementary Material

## 1 SUPPLEMENTARY DATA

The supplementary materials include:
1. Table S1. The results of association studies for AD patient modules.
2. FigS1. The pipeline for single-patient differential expression analysis.
3. FigS2. Distinct differential expression status among AD patients.
4. FigS3. Module analysis using the most observed differential expressed genes.
5. FigS4. Module patients reported more significant association than non-module patients.

## 2 SUPPLEMENTARY TABLES AND FIGURES

### 2.1 Tables

Table S1 The results of association studies for AD patient modules

### 2.2 Figures

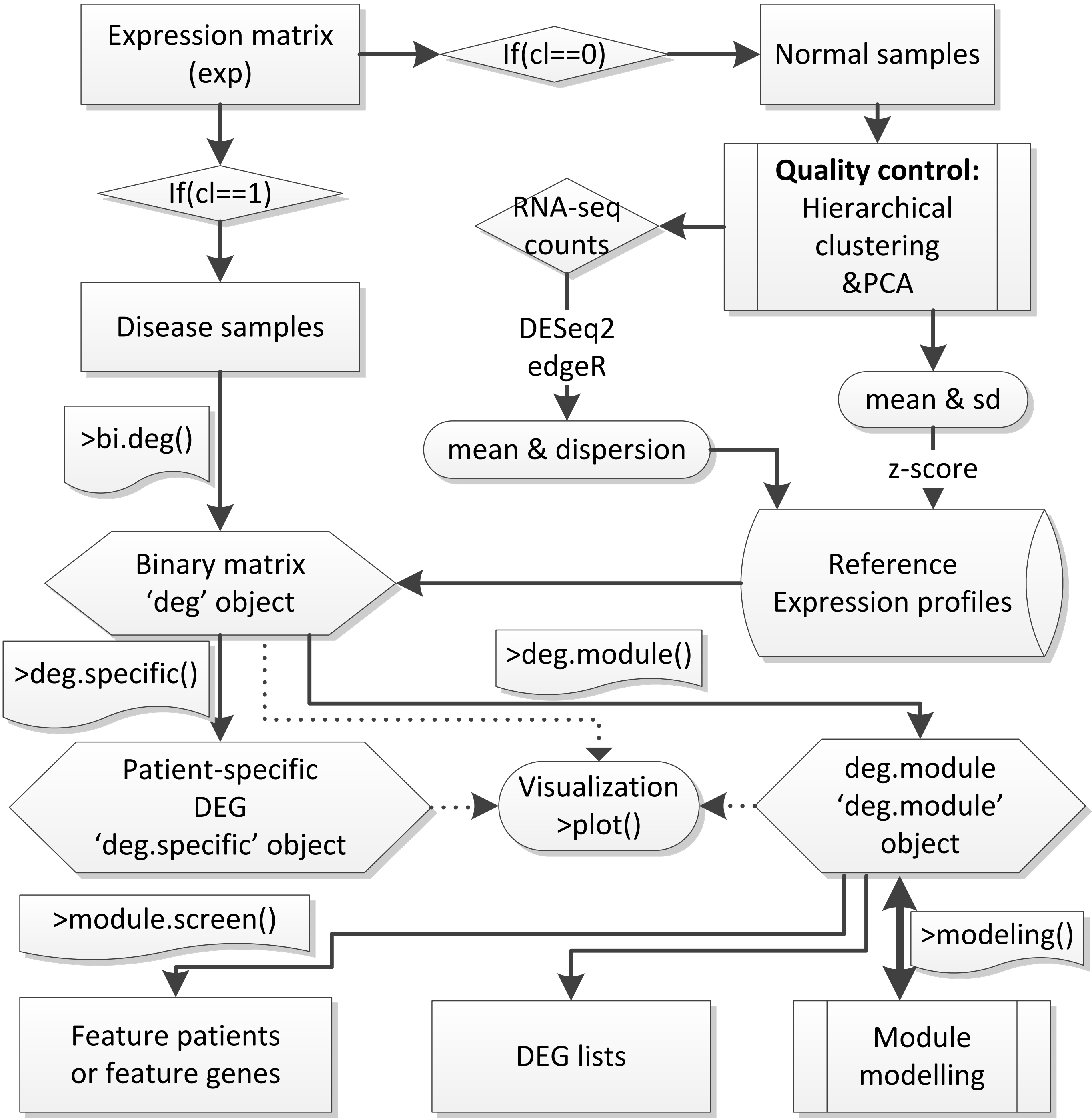


**Figure S1.** The pipeline for single-patient differential expression analysis.

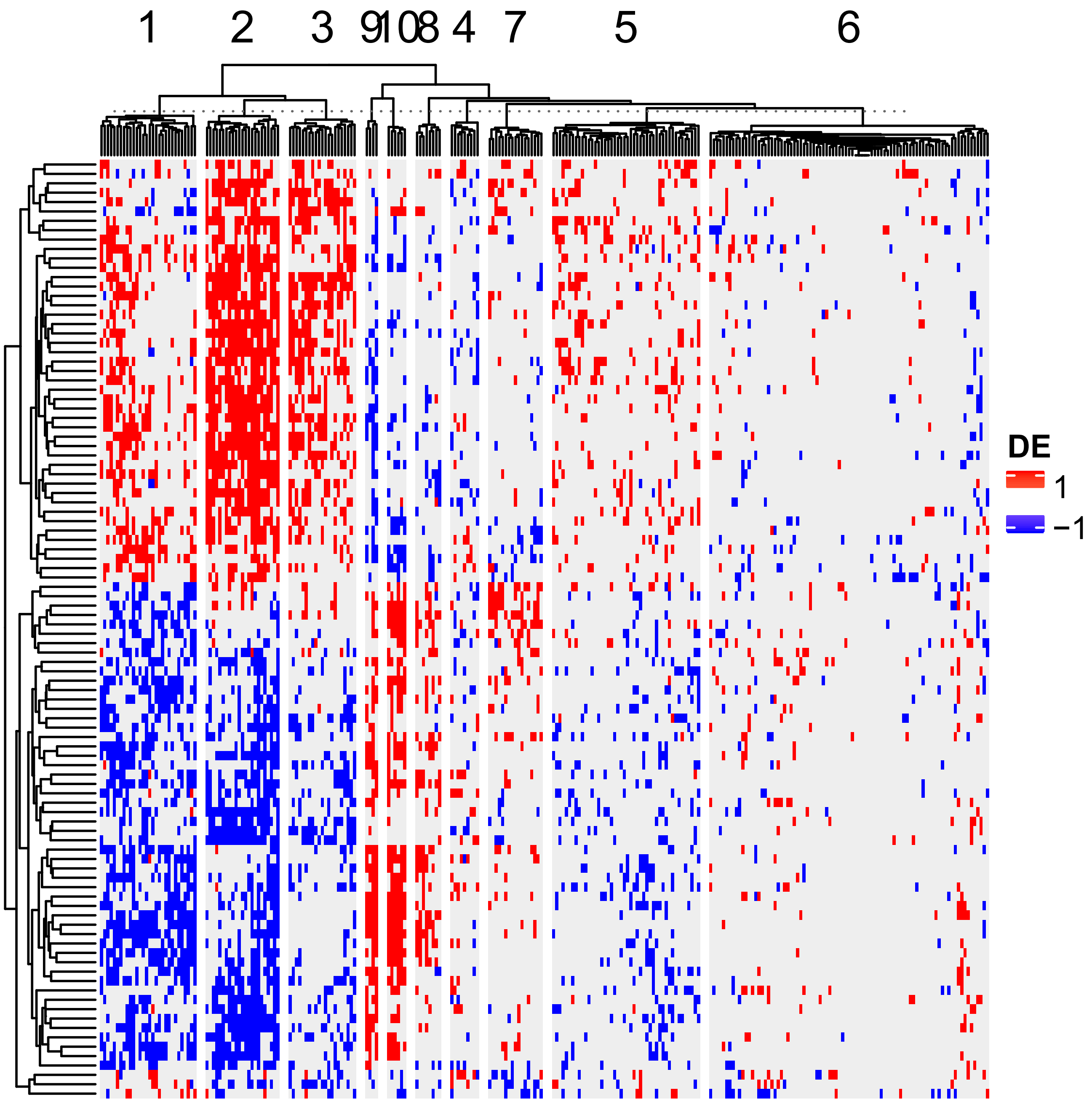


**Figure S2.** Distinct differential expression status among AD patients.

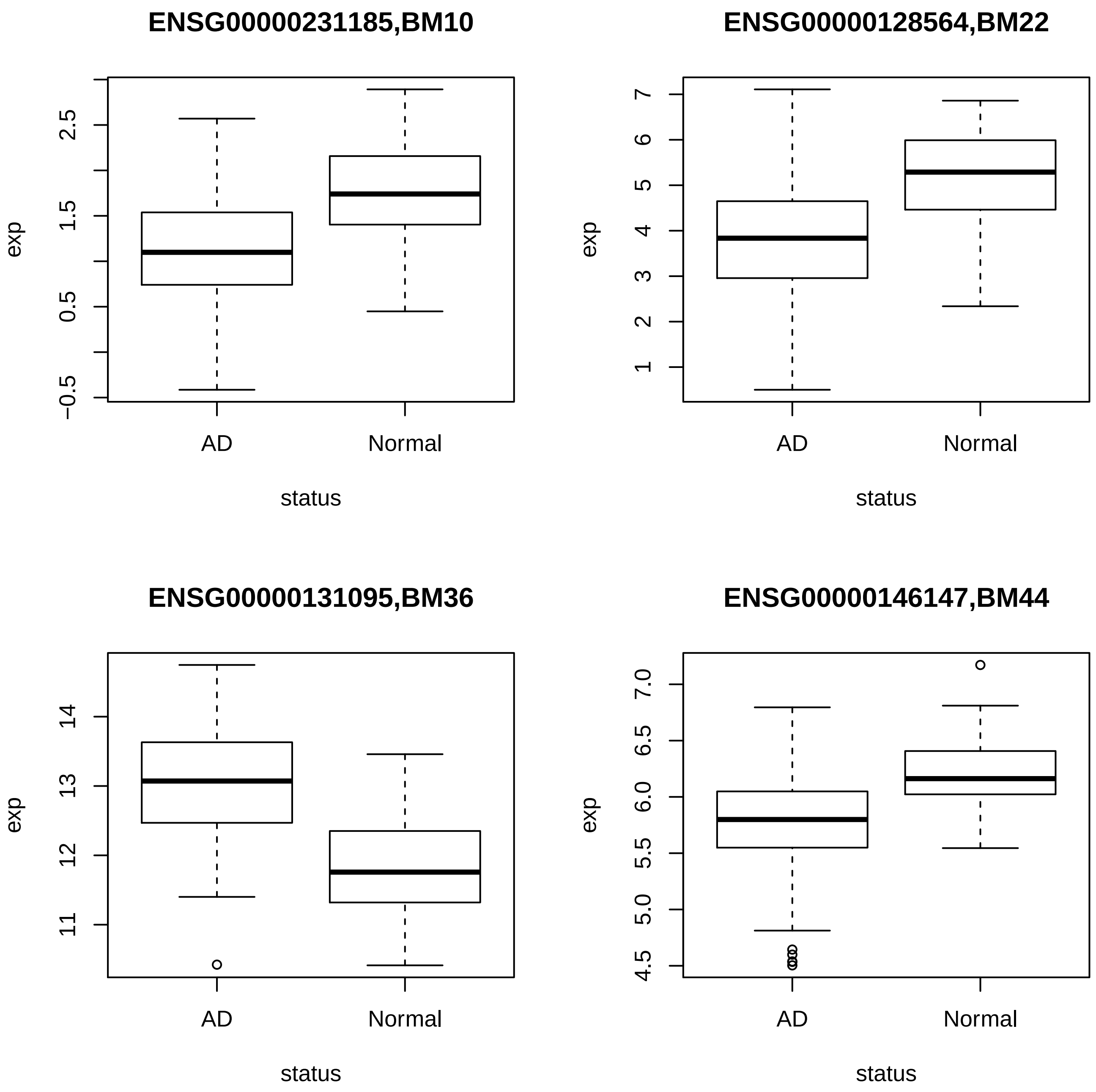


**Figure S3.** Module analysis using the most observed differential expressed genes.

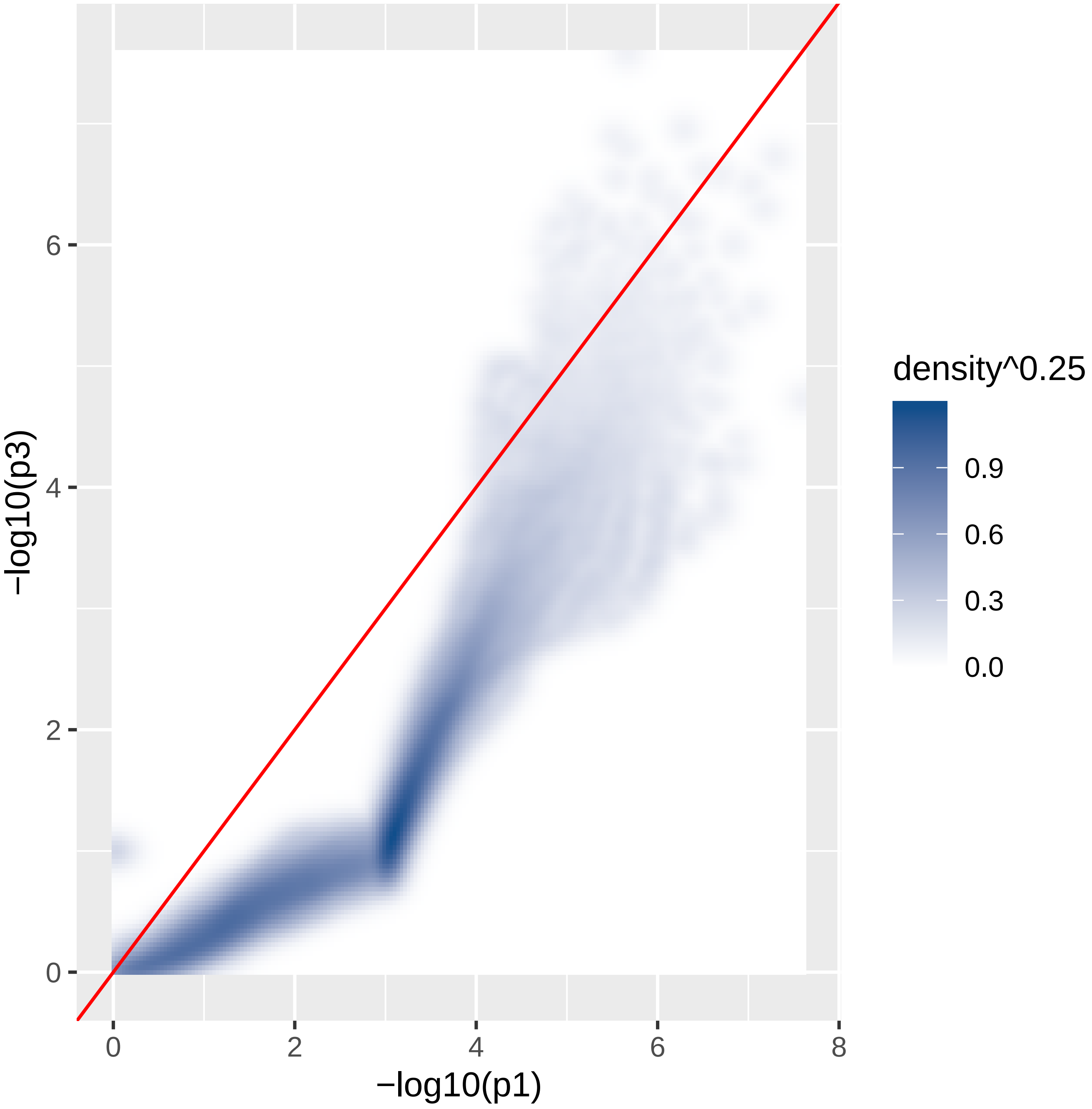


**Figure S4.** Module patients reported more significant association than non-module patients.
